# Sensitivity of Single-Molecule Array Assays for Detection of Clostridium difficile Toxins in Comparison to Conventional Laboratory Testing Algorithms

**DOI:** 10.1128/JCM.00452-18

**Published:** 2018-07-26

**Authors:** Alice Banz, Aude Lantz, Brigitte Riou, Agnès Foussadier, Mark Miller, Kerrie Davies, Mark Wilcox

**Affiliations:** a bioMérieux, Marcy L'Etoile, France; b Leeds Teaching Hospitals, Leeds, United Kingdom; c University of Leeds, Leeds, United Kingdom; Johns Hopkins University School of Medicine

**Keywords:** Clostridium difficile, ultrasensitivity

## Abstract

Guidelines recommend the use of an algorithm for the laboratory diagnosis of Clostridium difficile infection (CDI). Enzyme immunoassays (EIAs) detecting C. difficile toxins cannot be used as standalone tests due to suboptimal sensitivity, and molecular tests suffer from nonspecificity by detecting colonization. Sensitive immunoassays have recently been developed to improve and simplify CDI diagnosis. Assays detecting CD toxins have been developed using single-molecule array (SIMOA) technology. SIMOA performance was assessed relative to a laboratory case definition of CDI defined by positive glutamate dehydrogenase (GDH) screen and cell cytotoxicity neutralizing assay (CCNA). Samples were tested with SIMOA assays and a commercial toxin EIA to compare performance, with discrepancy resolution using a commercial nucleic acid-based test and a second cell cytotoxicity assay. The SIMOA toxin A and toxin B assays showed limits of detection of 0.6 and 2.9 pg/ml, respectively, and intra-assay coefficients of variation of less than 10%. The optimal clinical thresholds for the toxin A and toxin B assays were determined to be 22.1 and 18.8 pg/ml, respectively, with resultant sensitivities of 84.8 and 95.5%. In contrast, a high-performing EIA toxin test had a sensitivity of 71.2%. Thus, the SIMOA assays detected toxins in 24% more samples with laboratory-defined CDI than the high performing toxin EIA (95% [63/66] versus 71% [47/66]). This study shows that SIMOA C. difficile toxin assays have a higher sensitivity than currently available toxin EIA and have the potential to improve CDI diagnosis.

## Abstract (precis)

Guidelines recommend the use of an algorithm for the laboratory diagnosis of Clostridium difficile infection (CDI). Enzyme immunoassays (EIAs) detecting C. difficile toxins cannot be used as standalone tests due to suboptimal sensitivity, and molecular tests suffer from nonspecificity by detecting colonization.

## INTRODUCTION

Clostridium difficile infection (CDI) is a gastrointestinal disease caused by the Gram-positive bacteria Clostridium difficile (CD). This pathogen is responsible for pseudomembranous colitis and almost a quarter of all antibiotic-associated diarrhea (1). CDI has been shown to be common, serious, and costly (2), with varied disease severity that is associated with strain virulence and patient risk factors (3–6).

Guidelines exist that provide recommendations on how to diagnose and treat CDI patients (7–10). Accurate diagnosis is essential to control endemic spread and outbreaks and to provide appropriate treatment to the patient (11). Diagnostic tests detect the bacteria, bacterial components such as glutamate dehydrogenase (GDH), the toxins responsible for the disease, or genetic components such as the toxin genes. The presence of C. difficile toxins in a diarrheal fecal specimen may be the most reliable indicator of true CDI (12). However, toxin testing by current enzyme immunoassays (EIAs) is not suitable as a standalone test due to their lack of sensitivity. There are several commercially available EIAs for toxin detection, but none of them are as sensitive as the gold-standard method, the cell cytotoxicity neutralizing assay (CCNA), which measures toxin B cytotoxic activity (13, 14). Nevertheless, most laboratories do not perform the CCNA, as it is time consuming, requires cell-culture facilities and advanced technical skills, and has never been standardized. For these reasons, guidelines strongly recommend a two-stage testing approach (8, 10). This approach consists of the use of a highly sensitive assay with a high negative predictive value (thus ruling out CDI with a negative result), followed by secondary testing of positive specimens with an assay for the detection of C. difficile toxins. The highly sensitive assay recommended as a first step is either an EIA detecting GDH or a molecular assay detecting toxin genes. A positive result with molecular assay, if used as the only test for CDI, must be interpreted with caution, as it may be detecting carriage or colonization by a toxigenic strain in individuals who have diarrhea for other reasons (15). This is an important point to highlight, especially as the carriage rate can sometimes be quite high, up to 15% in adults (16, 17).

An automated one-step assay capable of detecting very small amounts of C. difficile toxins in a fecal sample could provide a significant improvement in the diagnosis of CDI. It has previously been shown that the single-molecule array (SIMOA) technology is able to detect a very low quantity of analyte in biological samples (18, 19). The development of sensitive assays by SIMOA for the detection of C. difficile A and B toxins in stool has shown promising results in comparison to toxigenic culture, CCNA, and molecular assay (20, 21). The objective of this study was to show that SIMOA toxin A and toxin B assays have a better analytical performance in comparison with one of the optimal EIAs currently available (13, 14).

## MATERIALS AND METHODS

### Clinical stool samples.

Frozen stool samples from Canadian (Jewish General Hospital, Montreal, Canada) and French (Charcot private hospital, Sainte-Foy-lès-Lyon, France) hospitals were used to determine the limit of detection (LOD) and the reproducibility of the two SIMOA toxin assays. For sensitivity and specificity studies, clinical samples (*n* = 240) were collected at the Leeds Teaching Hospitals NHS Trust in 2014 (Leeds, United Kingdom), and kept at −80°C. These samples were characterized for CDI status using a laboratory case definition defined by positive GDH screen and CCNA toxin assay. Informed consent was not required for the use of anonymized residual diagnostic material, and appropriate ethical approval was granted for use of the samples from Leeds by the University of Leeds.

### Sample preparation for SIMOA assays.

Frozen stool samples were thawed, then homogenized using a plastic spatula, and 50 to 100 mg of sample were transferred into an Eppendorf tube. The exact quantity of stool was determined by weighing the tube, and the volume of sample diluent added was adjusted to achieve a final dilution of 1/21 (mg/ml). Sample diluent consisted in Tris-buffered saline (pH 7.4) containing detergent and preservative. The tube contents were mixed using a vortex and centrifuged at 3,000 × *g* for 10 min. The supernatant was collected and tested for toxins.

### SIMOA toxin assays.

The SIMOA toxin A and toxin B assays were manufactured with the same raw materials as those previously described (20, 21), except for calibrators, antitoxin A antibodies (Ab), and detector. We used native toxins A and B, purified from C. difficile strain VPI 10463, toxinotype 0 (Native Antigen Company, Oxfordshire, United Kingdom), as calibrators; anti-toxin A antibodies from bioMérieux (bioMérieux, Marcy l'Etoile, France); and detector antibody directly conjugated to the enzyme β-galactosidase (Roche Diagnostics Corporation, Indianapolis, IN). Both toxin A and toxin B assays were composed of paramagnetic beads (Quanterix Corporation, Lexington, MA) coated with Ab directed against toxin A or B and detectors specific for each toxin. Assays were performed on a SIMOA HD-1 Analyzer (Quanterix Corporation, Lexington, MA) located at bioMérieux in France. Supernatants of specimens were tested in duplicate in each SIMOA run along with calibrators and controls. The controls were positive and negative stool samples characterized by French hospitals.

### Limits of detection (LOD) and limits of quantification (LOQ), repeatability, and reproducibility of SIMOA assays.

Six runs were performed, one run per day, for six days. Each run was composed of eight calibrators in duplicate, two negative samples, and 20 positive stool samples in quadruplet. The limit of blank (LOB) was determined as the 95th percentile of the blank sample distribution and calculated nonparametrically on 60 values (two negative samples tested in quadruplet and one sample diluent tested in duplicate per run on six runs). LOD and LOQ of the assays were determined by using the precision profile method on the six lowest positive samples. The precision profile characterizes the relationship between the standard deviation (SD) and the toxin concentrations. The mathematical model is a second-order polynomial equation: SD = *a*_0_ + *a*_1_*X* + *a*_2_*X*^2^ with *a*_0_, *a*_1_, and *a*_2_ corresponding to the parameters estimated for the second-order model and *X* being the concentration at the LOD level. LOD was calculated as LOD = LOB + Cp · SD, where Cp is a corrector factor of the SD, taking into account the total number of samples (*N*_tot_) and the number of replicates per sample (*K*),

$$Cp=\frac{1.645}{1-(\frac{1}{4(N_{total}-K)})}$$

The value of 1.645 represents the 95th percentile from the normal distribution for a risk, α, equal to 0.05. LOQ was defined as minimal concentration for a 10% coefficient of variation (CV) and calculated by resolution of the equation 0.1 LOQ = *a*_0_ + *a*_1_LOQ + *a*_2_ · LOQ^2^, with SD = CV · LOQ. LOB, LOD, and LOQ are expressed in pg/ml in pretreated samples (not corrected by dilution factor applied during stool pretreatment). The repeatability and reproducibility study was assessed using the SAS-Add-In 4.3 software (SAS Institute, Cary, NC) on 20 samples (24 values per sample).

### Repeatability of sample preparation for toxin detection.

Eighteen frozen clinical samples of different consistencies were selected, consisting of six liquid samples (Bristol stool chart type 7), six semiformed samples (Bristol stool chart types 5 and 6), and six formed stool samples (Bristol stool chart types 1 to 4). Samples were first homogenized with a plastic spatula. Eight individual sample preparations were then performed for each sample and tested in triplicate with the SIMOA toxin A assay to determine the variability in toxin concentration obtained between sample preparations. Due to the number of sample preparations done for each specimen, toxin quantification on freshly prepared samples was done with the SIMOA toxin A assay only. As the aim of this study was to evaluate the repeatability of sample preparation, we selected the SIMOA assay with the best repeatability to reduce/limit the variability due to the SIMOA assay and instrument.

### Clinical sensitivity and specificity of SIMOA toxin assays.

The clinical threshold of the SIMOA assays was determined with a ROC (receiver operating characteristics) curve. This curve was drawn with Analyse-it software (Analyse-it Software, Ltd., Leeds, United Kingdom) using the concentration obtained for each diluted sample and the patient's CDI status, as determined by laboratory case definition. The clinical thresholds were chosen according to the Youden's index value given by the software. The sensitivity and specificity obtained for SIMOA assays using the selected clinical thresholds were determined by comparison to the laboratory case definition. A patient was diagnosed with CDI if the fecal sample was found positive by GDH and CCNA assays, while a patient was considered CDI negative if the sample was found to be negative by GDH or CCNA assay. The sensitivity and specificity are given as percentages with the 95% confidence interval (95% CI) values.

### Comparison to a commercially available immunoassay.

All samples were tested with the commercially available C. difficile Tox A/B II assay (Techlab, Blacksburg, VA), a fecal C. difficile toxin A and toxin B enzyme immunoassay, according to the manufacturer's instructions. The C. difficile Tox A/B II assay was performed in parallel with the SIMOA assay, using the same aliquot. One-third of the samples had an additional freezing/thawing cycle before testing with the C. difficile Tox A/B II assay.

### Toxin gene detection by molecular assay.

The Xpert C. difficile assay (Cepheid Inc., Sunnyvale, CA) was performed using the Cepheid GeneXpert Systems. Sixty of the 96 GDH-positive CCNA-negative samples were tested with the Xpert C. difficile assay by the Leeds laboratory as part of an internal evaluation. At bioMérieux, the testing of the 36 GDH-positive CCNA-negative samples was completed, as well as additional testing for samples that gave discordant results between CCNA and the SIMOA assay.

### Cell cytotoxicity neutralizing assay (CCNA).

CCNA was performed at bioMérieux (bMx CCNA), on fecal samples that showed discordant results between the CCNA obtained by the Leeds laboratory and SIMOA, to verify that the toxin was present and functional in the samples. Vero cells (ATCC CCL-81) were inoculated at 30,000 cells/well on the day prior to the test. Samples were solubilized in minimum essential medium (MEM) with 1% fetal calf serum and 1% gentamicin, streptomycin, penicillin, and amphotericin B (Thermo Fisher Scientific, Waltham, MA) at 1/6 dilution and centrifuged at 3,000 × *g* for 10 min. The supernatant was then diluted 1/10 and filtered at 0.45 μm, and three other successive dilutions were done to achieve the following final dilutions: 1/120; 1/1 200; 1/12,000; and 1/120,000. Positive and negative controls (stool samples and media) were tested in parallel to the samples. A neutralizing antitoxin B Ab (Meridian Life Science, Memphis, TN) was used to verify that the cytotoxic activity was caused by toxin B. The plate was examined at 24 and 48 h for cell rounding. In terms of analytical sensitivity of the bMx CCNA assay, we have observed high cytotoxic activity using native toxin A at 250 pg/ml, moderate activity at 100 pg/ml, low activity at 20 pg/ml, and no activity at 10 pg/ml. For native purified toxin B the cytotoxic activity was high at 5 pg/ml, moderate at 1.5 pg/ml, low at 0.2 pg/ml, and nonexistent at 0.02 pg/ml.

The differences with the method used at the Leeds Hospital (Leeds CCNA) were the following: the origin of Vero cells (European Collection of Authenticated Cell Cultures, Wiltshire, United Kingdom), the diluent used to dilute the stool sample (phosphate-buffered saline), the preparation of the sample (filtration of sample supernatant), the anti-toxin antibody (C. sordelli antitoxin, Prolab, United Kingdom), and the tested dilutions (1/100 and 1/1,000).

## RESULTS

### Limits of detection and quantification, repeatability, and reproducibility of SIMOA assays.

**FIG 1:**
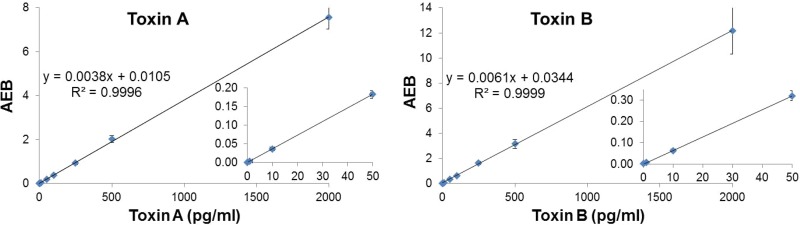
Assay measuring range; calibration curves of SIMOA toxin A and toxin B assays. The graphs represent the mean of average enzyme per bead (AEB) values, corresponding to SIMOA signal output, obtained with SIMOA toxin A and toxin B assays for eight calibrators (purified native toxins), which ranged from 0 to 2000 pg/ml. Means and standard deviations for each calibrator correspond to duplicates of eight individual runs on an SIMOA HD-1 analyzer. Regression equations and coefficients of determination (*R*^2^) of the slopes are indicated on each figure.

The toxin A and toxin B concentrations in diluted fecal specimen were determined using calibration curves ranging from 1 to 2,000 pg/ml (Fig. 1). The LOBs obtained with the negative samples were 0.4 and 2.4 pg/ml for toxins A and B, respectively, while the LODs of SIMOA toxin A and toxin B assays were 0.6 and 2.9 pg/ml (Table 1), respectively. LOQ equaled LOD for the toxin B assay, as CVs of samples in this concentration range were below 10%. Intra-assay and interassay CVs for samples above the LOQ were below 10% for the SIMOA assays, except for 1 sample having an interassay CV of 10.4% for the SIMOA toxin A assay and 2 samples having interassay CVs of 11.3% and 14% for the SIMOA toxin B assay. Table 2 shows results obtained for 4 representative samples covering the measuring range of the 20 tested.

**TABLE 1:** LOB, LOD, and LOQ of both SIMOA toxin assays in pretreated samples^*a*^

Test	LOB (pg/ml)	LOD (pg/ml)	LOQ (pg/ml)
SIMOA toxin A	0.4	0.6	1.7
SIMOA toxin B	2.4	2.9	2.9

a LOB, limit of blank; LOD, limit of detection; LOQ, limit of quantification.

**TABLE 2:** Repeatability and reproducibility of SIMOA toxin assays

Sample	Assay for toxin A			Assay for toxin B		
	Mean toxin (pg/ml)	Intra-assay CV*^a^* (%)	Interassay CV (%)	Mean toxin (pg/ml)	Intra-assay CV (%)	Interassay CV (%)
1	3.7	4.9	5.4	3.2	7.1	9.8
2	12	3.4	4.9	10.4	3.6	11.3
3	32	2.2	3.8	28	4	7.4
4	633	3.6	5.8	687	0	7.8

### Repeatability of sample preparation for toxin detection.

To evaluate the repeatability of the sample preparation method for toxin detection, clinical specimens from different consistencies, ranging from liquid to formed stools, were used. A good repeatability of toxin quantification was observed for all six liquid samples tested (Bristol stool chart type 7). Table 3 shows the results obtained for toxin A (CVs below 10% for the eight independent preparations of each sample). For semiformed (Bristol stool chart types 5 and 6) and formed samples (Bristol stool chart types 1 to 4), four of the six samples tested had a CV of less than 10%. The two formed samples with CVs greater than 10% were classified as hard stool using the Bristol stool chart (type 1 and 2), while the two semiformed samples had a consistency that was difficult to homogenize. The issue of obtained repeatability with these samples was directly correlated to the stool consistency (hard stool or fibrous/filamentous samples) and the difficulty of efficiently homogenizing the sample in the diluent.

**TABLE 3:** Repeatability of sample preparation for toxin A detection

Sample	Liquid samples		Semiformed samples		Formed samples	
	Toxin A (pg/ml)	CV (%)	Toxin A (pg/ml)	CV (%)	Toxin A (pg/ml)	CV (%)
1	74	2.4	70	7.2	33	8
2	176	6.4	154	2.1	62	11.6
3	198	4.5	171	7.1	134	5
4	450	2.1	450	18.4	737	5.4
5	1,020	7.2	459	38.4	1,413	19.7
6	2,365	4.7	692	6.4	2,788	2.5

### Sensitivity and specificity of SIMOA toxin assays.

The sensitivity and specificity of SIMOA toxin assays were evaluated with clinical samples from 240 patients, consisting of 126 females and 114 males of ages ranging from 7 to 86 years, with a median of 74 years. Using a laboratory case definition, 66 patients were diagnosed with CDI while 174 patients were CDI negative.

**FIG 2:**
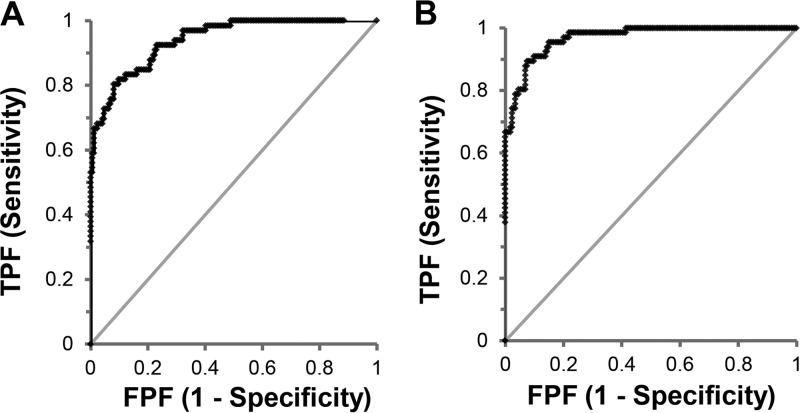
Receiver operating characteristic (ROC) curves obtained for SIMOA toxin A (A) and toxin B (B) assays. The ROC curves of SIMOA toxin A and toxin B assays are created by plotting the true-positive rate against the false-positive rate at various threshold settings, using the concentration obtained for each pretreated sample and the CDI status of the patient (*n* = 240).

**FIG 3:**
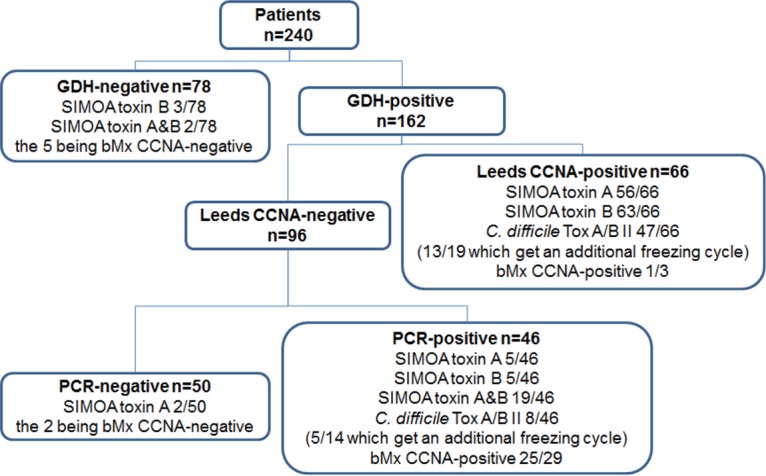
Number of samples given a positive result with SIMOA assays and the C. difficile TOX A/B II, according to Leeds laboratory results. The figure shows the number of patients tested and the number of positive and negative results obtained with GDH screen, Leeds CCNA, and PCR assay. For each group, the number of samples positive by SIMOA toxin A assay, SIMOA toxin B assay or both SIMOA assays and with C. difficile TOX A/B II are indicated. The results obtained with bioMérieux's CCNA (bMx CCNA) for discordant results are also indicated, as well as the number of samples which got an additional freezing cycle.

Figure 2 shows the ROC curves of the toxin concentration relative to the CDI status of the patients. The areas under the curve (AUC) obtained for SIMOA toxin A and toxin B assays were 0.941 and 0.969, respectively. The selected clinical thresholds were 22.1 and 18.8 pg/ml for toxin A and toxin B, respectively, giving sensitivities of 84.8 and 95.5% and specificities of 83.9 and 83.3% compared to the laboratory case definition (Table 4). By comparison, the C. difficile Tox A/B II showed a lower sensitivity of 71.2% and a higher specificity of 95.4% compared to the laboratory case definition. The sensitivity and specificity of the combined SIMOA toxin assays were determined by considering a sample positive if at least one of the two assays gave a positive result. Accordingly, the sensitivity of combined SIMOA assays corresponds to the sensitivity of the toxin B assay, which has the higher sensitivity of the two. The specificity of the combined assays is lower than the specificity of each assay due to the fact that 8 samples were positive for toxin B only and 7 for toxin A only (Fig. 3).

**TABLE 4:** Sensitivity and specificity of SIMOA toxin assays compared to CDI status^*a*^

Test	Sensitivity (% [95% CI]) (*n* = 66)	Specificity (% [95% CI]) (*n* = 174)
SIMOA toxin A	84.8 (73.9–92.5)	83.9 (77.6–89)
SIMOA toxin B	95.5 (87.3–99.1)	83.3 (77–88.6)
SIMOA toxin A + B	95.5 (87.3–99.1)	79.3 (72.5–85.1)
C. difficile Tox A/B II	71.2 (58.8–81.7)	95.4 (91.1–98)

a Totals of 66 GDH- and CCNA-positive samples and 174 GDH- and/or CCNA-negative samples. CI, confidence interval.

The low specificity of both SIMOA assays in comparison to the laboratory case definition is mainly due to the 29 SIMOA toxin-positive results among the 46 GDH- and PCR-positive and CCNA-negative samples (Fig. 3). Some of those samples (*n* = 8/29; 28%) were also positive by the C. difficile Tox A/B II assay. These SIMOA toxin-positive samples were retested inhouse with bMx CCNA and 86% (25/29, Fig. 3) were found positive at a low dilution factor. Discrepancy in the CCNA results between the Leeds laboratory and the bioMérieux inhouse results may be explained by several facts, including the different methods used (cells, antibodies, sample dilution, and neutralizing antibody) and the visual analysis of cytotoxicity. Nevertheless, analysis of discordant results with bMx CCNA showed 11 SIMOA false-positive (toxin A and/or B) results (Fig. 3).

**FIG 4:**
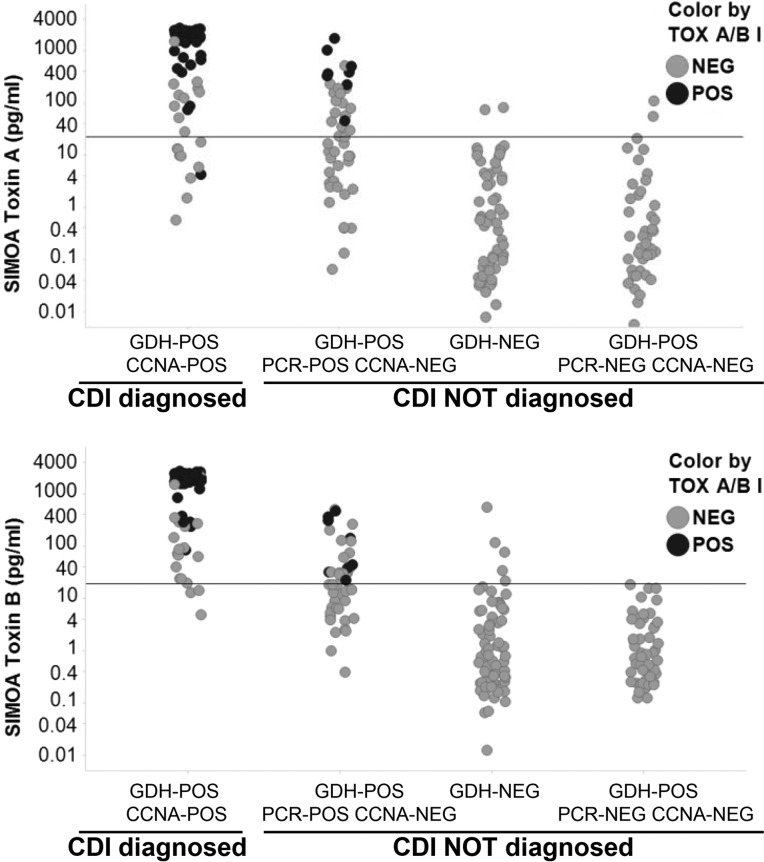
SIMOA toxin A and toxin B assay results according to laboratory case definition of CDI and comparison to C. difficile TOX A/B II assay results. Toxin A and toxin B concentrations measured with SIMOA assays in each diluted sample (*n* = 240) are plotted according to the CDI status of the patient, CDI diagnosed (GDH- and Leeds CCNA-positive sample) or CDI not diagnosed (GDH- and/or Leeds CCNA-negative sample). The positivity thresholds of SIMOA toxin A and toxin B assays are indicated by a line on each figure (toxin A, 22.1 pg/ml; toxin B, 18.8 pg/ml). Each circle corresponds to a quantity of toxin determined by SIMOA assays, and dark circles represents the sample which are positive by the C. difficile TOX A/B II assay. Toxin concentrations in the graph are not corrected by the dilution factor.

The sensitivity of the SIMOA toxin B assay was higher than the sensitivity of the toxin A assay, as expected, given that the comparison was done with CCNA, an assay more sensitive to toxin B (Table 4). Toxin A was not detected in 10 CCNA-positive samples, while toxin B was not detected in only 3 of these 10 samples (Fig. 3 and 4). Repeat inhouse bMx CCNA for these 3 samples gave a negative result for 2 of them, suggesting a potential degradation of the toxins. By comparison, the C. difficile Tox A/B II assay was negative in 19 of the 66 CCNA-positive samples. The SIMOA toxin B assay detected toxins in 84% (16/19) of EIA-negative samples, resulting in 24% (16/66) more samples determined as positive (Fig. 3 and 4).

For a third of the samples, an additional freezing/thawing cycle occurred before testing with the C. difficile Tox A/B II assay. Thirteen of the 19 CCNA-positive samples, which got an additional freezing/thawing cycle, gave a positive result with the C. difficile Tox A/B II assay, and 6 were found negative, one also being negative with SIMOA. Among the 56 negative samples having an additional freezing/thawing cycle, four gave a positive result with C. difficile Tox A/B II assay. These samples were also found positive with the Xpert C. difficile assay and inhouse bMx CCNA. The sensitivity and specificity calculated on the 165 samples that did not get an additional freezing/thawing cycle, gave equivalent results to the sensitivity and specificity determined on all the samples (Table 5).

**TABLE 5:** Sensitivity and specificity of SIMOA toxin assays compared to CDI status using samples that did not get an additional freezing/thawing cycle^*a*^

Test	Sensitivity (% [95% CI]) (*n* = 47)	Specificity (% [95% CI]) (*n* = 118)
SIMOA toxin A	85.1 (71.7–93.8)	85.6 (77.9–91.4)
SIMOA toxin B	95.7 (85.5–99.5)	85.6 (77.9–91.4)
SIMOA toxin A + B	95.7 (85.5–99.5)	80.5 (72.2–87.2)
C. difficile Tox A/B II	72.3 (57.4–84.4)	96.6 (91.5–99.1)

a Totals of 47 GDH- and CCNA-positive samples and 118 GDH- and/or CCNA-negative samples.

## DISCUSSION

EIAs detecting toxins have been developed to facilitate diagnosis of CDI, but none of the commercially available EIAs has sensitivity equivalent to that of CCNA (13). On the other hand, molecular assays detecting C. difficile genes (including toxin genes) have shown higher sensitivity in comparison that of to CCNA but have been criticized for detecting colonized patients having diarrhea for other reasons (15). Furthermore, work done by Polage et al. and Planche et al. have shown that patients outcome differs according to the testing method. The presence of toxin in the stool strongly correlates with duration of the symptoms and with disease severity, whereas the presence of toxin gene alone did not (12, 22). These results suggest that a sensitive automated assay for the detection and quantification of C. difficile toxins in stool could be the most appropriate method for CDI diagnosis. Assays detecting toxins A and B have been recently developed using the SIMOA technology and showed a sensitivity of toxin B assay equivalent to that of CCNA and a specificity close to that of molecular assays and toxigenic culture (20).

We have developed SIMOA toxin A and toxin B assays according to Quanterix methods, using raw materials similar to the ones described by Song et al. (21), and showed that these assays have a better analytical performance than one of the best EIAs commercially available. Our SIMOA toxin A and toxin B assays have LOD of 0.6 and 2.9 pg/ml in pretreated fecal aliquots, respectively, corresponding to 0.013 and 0.061 pg/mg of toxin in original stool samples. These results are slightly different from the ones previously described (LOD, 0.45 and 1.5 pg/ml, corrected for the dilution factor) (21). We believe that this is probably related to the native toxins, which are used as calibrators, and the method used to define these values. Nevertheless, with our clinical cutoffs of 22.1 and 18.8 pg/ml for toxin A and toxin B assays in pretreated samples, we observed similar sensitivity and specificity to the ones published in Song et al. (21), even though the patient populations were not characterized in the same way. In our study, the sensitivity of the SIMOA toxin B assay in comparison to the laboratory case definition was 95.5%, while the sensitivity described by Song et al. (21) was 100% in comparison to that of CCNA. The specificities of our SIMOA toxin A and toxin B assays, compared to that of the Leeds CCNA, were 83.9% and 83.3%, respectively, while Song et al.'s results were 84% and 87%. In both our and Song et al.'s studies, a number of PCR-positive CCNA-negative samples were found positive by the SIMOA toxin assays. Some of the samples also gave a positive result by the C. difficile Tox A/B II assay. As previously mentioned by Song et al. (21), this not only brings into question the sensitivity of CCNA, but also the accuracy, reproducibility, and robustness of this poorly standardized assay, which can be performed on different cell lines, using various dilutions, and sometimes without antitoxin confirmation. The quantity of toxin measured by SIMOA assay in these samples was low in comparison to the quantity in other CDI patients, also observed by Song et al. The methodology of EIAs and CCNAs are not the same. Detection of C. difficile toxin B presence is physically determined by EIA, whereas CCNA results demonstrate the results of toxin B activity. One potential explanation for the SIMOA-positive CCNA-negative samples may be the preanalytical loss of toxin B activity, whereas toxins remain detectable by immunoassay.

At this time, we do not understand the true meaning for patients when a low toxin concentration is detected. Highly sensitive toxin assays may suffer from the same specificity problem as highly sensitive molecular tests, which can lead to the erroneous identification of uninfected individuals as CDI patients (23). Since it is considered unnecessary to treat colonized patients (24), we face a clinical quandary about how to manage individuals with low fecal toxin concentrations—are they colonized or infected? Whether we can distinguish infected and colonized patients with SIMOA C. difficile toxin assays, based on toxin concentrations, is currently unknown. Additional epidemiologic and interventional studies are required to answer this question.

The limits of our study are the lack of clinical confirmation of CDI status, the number of samples tested, the comparison to other EIAs detecting toxins, and the fact that an additional freezing/thawing cycle occurred for some samples prior to testing with the C. difficile Tox A/B II assay. It is known that a freezing/thawing cycle is not recommended for toxin stability. Nevertheless, it is important to point out that toxins were detected by C. difficile Tox A/B II assay in samples that had an additional freezing/thawing cycle, and that the sensitivity and specificity values were not different without these samples. Finally, clinical correlation studies of CDI presentation and outcome with a large number of samples will be required for determination of the diagnostic accuracy of these assays.

In our comparison of the SIMOA assays and the C. difficile Tox A/B II assay, we observed that the SIMOA toxin A and toxin B assays have higher sensitivities than the C. difficile Tox A/B II assay (84.8 and 95.5% versus 71.2%). The use of the SIMOA assays allowed the detection of toxin-positive samples that would have been missed by the commercial EIA. In comparison to the analytical sensitivity of EIAs determined in diluted supernatant (without correction by dilution factor), we described a LOD of toxin B of 2.9 pg/ml, corresponding to 0.061 pg/mg of toxin in the stool. Hence, C. difficile toxin detection using SIMOA technology has the potential to improve CDI diagnosis. The SIMOA assay could replace current EIAs for laboratories that do not have the skills and capacity to perform CCNA and would increase the specificity of positive results compared with that of current molecular tests.
